# Genetic Polymorphism of Delta Aminolevulinic Acid Dehydratase (*ALAD*) Gene and Symptoms of Chronic Mercury Exposure in Munduruku Indigenous Children within the Brazilian Amazon

**DOI:** 10.3390/ijerph18168746

**Published:** 2021-08-19

**Authors:** Jamila Alessandra Perini, Mayara Calixto Silva, Ana Claudia Santiago de Vasconcellos, Paulo Victor Sousa Viana, Marcelo Oliveira Lima, Iracina Maura Jesus, Joseph William Kempton, Rogério Adas Ayres Oliveira, Sandra Souza Hacon, Paulo Cesar Basta

**Affiliations:** 1 Laboratório de Pesquisa de Ciências Farmacêuticas (LAPESF), Centro Universitário Estadual da Zona Oeste (UEZO), Av. Manuel Caldeira de Alvarenga, 1.203, Rio de Janeiro 23070-200, RJ, Brazil; jamilaperini@yahoo.com.br (J.A.P.); mayaracx_2010@hotmail.com (M.C.S.); 2 Laboratório de Educação Profissional em Vigilância em Saúde, Escola Politécnica de Saúde Joaquim Venâncio, Fundação Oswaldo Cruz (EPSJV/Fiocruz), Av. Brasil, 4365-Manguinhos, Rio de Janeiro 21040-900, RJ, Brazil; anacsvasconcellos@gmail.com; 3 Centro de Referência Professor Hélio Fraga, Escola Nacional de Saúde Pública, Fundação Oswaldo Cruz (CRPHF/ENSP/Fiocruz), Estrada de Curicica, 2000-Curicica, Rio de Janeiro 22780-195, RJ, Brazil; paulovictorsviana@gmail.com; 4 Seção de Meio Ambiente, Instituto Evandro Chagas, Secretaria de Vigilância em Saúde, Ministério da Saúde (SEAMB/IEC/SVS/MS), Rodovia BR-316 km 7 s/n-Levilândia, Ananindeua 67030-000, PA, Brazil; marcelolima@iec.gov.br (M.O.L.); iracinajesus@iec.gov.br (I.M.J.); 5 Faculty of Medicine, Imperial College London, Medical School Building, St Mary’s Hospital, Norfolk Place, London W2 1PG, UK; kemptonj@hotmail.com; 6 Faculdade de Medicina, Universidade de São Paulo—USP, São Paulo 01246-903, SP, Brazil; roger.adas.doc@gmail.com; 7 Departamento de Endemias Samuel Pessoa, Escola Nacional de Saúde Pública, Fundação Oswaldo Cruz (ENSP/Fiocruz), Rua Leopoldo Bulhões, 1480-Manguinhos, Rio de Janeiro 21041-210, RJ, Brazil; sandrahacon@gmail.com

**Keywords:** mercury exposure, *ALAD*, genetic polymorphism, neurotoxicity, environmental health, indigenous people

## Abstract

Genetic polymorphisms involved in mercury toxicokinetics and toxicodynamics may be associated with severe mercury toxicity. This study aimed to investigate the impact of an *ALAD* polymorphism on chronic mercury exposure and the health situation of indigenous children from the Brazilian Amazon. One-hundred-and-three indigenous children (under 15 years old) were included and genotyped (rs1800435) using a TaqMan validated assay. The mean age was 6.6 ± 4.5 years old, 60% were female, 49% presented with anemia, and the mean hair mercury concentration was 7.0 ± 4.5 (1.4–23.9) µg/g, with 49% exceeding the reference limit (≥6.0 µg/g). Only two children were heterozygous *ALAD*, while the others were all wild type. Minor allele frequency (*ALAD G*) and heterozygous genotype (*ALAD CG*) were 1% and 2%, respectively. The two children (12 and 14 years old) with the *ALAD* polymorphism had mercury levels above the average as well as had neurological symptoms related to chronic mercury exposure, such as visual field alterations, memory deficit, distal neuropathy, and toe amyotrophy. Both children also reported frequent consumption of fish in the diet, at least three times a week. In conclusion, our data confirm that an *ALAD* polymorphism can contribute to mercury half-life time, harmful effects, and neuropsychological disorders in indigenous children with chronic mercury exposure to gold mining activity.

## 1. Introduction

Our society has long made use of Mercury (Hg) in industry and technology. However, mercury exposure is a global public health concern due to its adverse effects on human health and the environment. The three forms of mercury, elemental, inorganic, and organic, induce toxic effects with different transport and metabolic mechanisms [1,2]. The organic form of mercury, methylmercury (MeHg), is the most toxic to humans due to toxicokinetic features: fast absorbed; extensively distributed to all tissues, including the hematoencephalic and placental barriers; and slowly eliminated. In addition, mercury exposure has already been associated with developmental disorders and impaired growth, as well as behavioral, immunological, hormonal, reproductive, and neurological changes [1,3,4,5].

Polymorphisms in genes that encode proteins involved in mercury toxicokinetics and toxicodynamics may be valuable for a personalized response to detrimental mercury toxicity, especially among vulnerable population groups with significant mercury exposure [6,7]. The indigenous communities of the Brazilian Amazon are one of these vulnerable groups owing to the high levels of MeHg found in the food staples they rely on, including fish and hunted animals. This makes it environmentally relevant [5,8].

The delta aminolevulinic acid dehydratase (*ALAD*) enzyme is required in heme synthesis and plays an important role in metal toxicokinetics, mainly transporting metals through the body. *ALAD* polymorphism rs1800435 in exon 4 (chromosome 9q34) is characterized by a *C > G* allele change (*177 C > G*), which results in a Lys-Asn variation in position 59 of the protein. This Lys-Asn variation, therefore, creates a different functional isozyme with different protein binding affinity for metals [9,10]. This *ALAD* polymorphism was associated with higher Hg concentrations in blood and lower enzyme activity than non-variant subjects from riverside Amazonian communities [11]. In addition, it has already been observed that in similar mercury exposures, children have a higher susceptibility to adverse neurological effects than adults [7,12]. Neuropsychological disturbances were associated with exposure to mercury in children from the Amazon region [13].

Thus, the objectives of this study were (1) to identify the presence of this *ALAD* polymorphism in the Munduruku indigenous communities of the Brazilian Amazon and (2) to evaluate the effects of this polymorphism on the chronic exposure to mercury and on the health situation of them.

## 2. Materials and Methods

### 2.1. Study Population and Clinical Evaluation

This study is part of a major project entitled “Mercury exposure in Munduruku indigenous communities from Brazilian Amazon: Methodological background and an overview of the principal results” [14]. Here, a case study was carried out involving indigenous children (under 15 years old) from *Sawré Muybu*, *Poxo Muybu*, and *Sawré Aboy* villages (Munduruku people of the middle Tapajós River) between October and November 2019. The National Ethics Committee of Human Research approved this study (protocol number 65671517.1.0000.5240). After presenting the research protocol to the community and clarifying doubts, the guardian of each participant provided written informed consent and answered a questionnaire, as previously described [14]. All residents under 15 years old were invited to participate in the study and there was no refusal. Furthermore, no probabilistic sampling methods were used to select the participants.

The research team collected data through (i) home visits and interviews with participating families; (ii) clinical and laboratory evaluation; (iii) collection of hair samples for mercury exposure analysis; and (iv) collection of epithelial cells from the oral mucosa collected by swab for DNA extraction and genotyping analysis of an *ALAD* polymorphism.

The body mass index (BMI) was calculated as the weight (kg) divided by the square of height (m^2^) for those over 6 years old (*n* = 60), and the Z-scores (BMI for age) for children from 0 to 5 years old, were calculated according to the World Health Organization [15]. The hemoglobin levels were assessed using the Hemocue device, and anemia was considered when the levels were below 11.5 g/dL. The blood pressure of participants over 12 years of age (*n* = 22) was measured twice using an automatic pulse blood pressure monitor (Omron Model Hem-631INT). The means of the two measures of systolic blood pressure (SBP) and diastolic blood pressure (DBP) were used to classify the participants [16], and hypertension was considered when SBP values were ≥140 mm Hg and DBP were ≥90 mm Hg.

### 2.2. Neurological Assessment

Participants aged 12 years or older (*n* = 15) underwent a systematized neurological examination protocol, specially developed for this research. The assessments were carried out by one of the authors (RAAO), and the other two neurologists (BDP, BHR) familiarized with neurological semiology and trained in the application of the protocol and the assessment scales. We assessed the balance and gait, coordination, Achilles deep tendon reflex test, and complete cranial nerve evaluation, including visual campimetry by confrontation, pupillary and extrinsic ocular motricity, facial motility and sensitivity, and elevation of the palate and vomiting reflex. Moreover, we tested the painful nociceptive as well as the tactile and thermal sensitivity to cold in the upper and lower limbs in the respective proximal and distal segments with standardized instruments. For cognitive assessments, we applied the Short Battery of Cognitive Screening Instrument [17], which includes the following domains: (i) immediate memory; (ii) learning; (iii) interference task; and (iv) long-term memory. Verbal Fluency Test in the animal category [18] and Stick Design Test [19] were also used to assess cognition. For further details on neurological assessment, see Oliveira et al. (2021) [20].

A neurodevelopment assessment was performed with the Denver II neurodevelopment screening test in children aged 0 to 6 years (*n* = 56), without morphological changes. The Denver II test assesses and identifies children at risk for developmental impairment. The test is divided into four areas: (i) personal-social: aspects of the child’s socialization inside and outside the family environment; (ii) fine motor skills: hand-eye coordination and manipulation of small objects; (iii) language: production of sounds, ability to recognize, understand, and use language; (iv) gross motor skills: body motor control, sitting, walking, jumping, and the other movements performed by large muscles, as previously described [21].

### 2.3. Hair Mercury Analysis

Hair samples (*n* = 102) were collected from the occipital area and mercury speciation analysis was performed based on a previously optimized procedure [14,22]. The hair mercury detection method was previously described [22,23,24]. For quality assurance and control of the method we used: (i) a 6-point calibration curve with concentrations ranging from 0.4 to 4 ng/g; (ii) the Human Hair Certified Reference Material (IAEA-86), whose average recovery rate was 101% (*n* = 8, recovery ranging from 83.4 to 106.6%) from the International Atomic Energy Agency; and (iii) the relative standard deviation (RSD) of 8.32%. The detection and quantification limits (LOD/LOQ) obtained were 0.0083 ng/mg and 0.027 ng/mg, respectively. Sample replicates (*n* = 10), whose RSD was 2.49%, were also randomly selected. The level of ≥6.0 µg/g of mercury in hair samples was considered as a health risk limit indicator, as previously described in the Amazon region [23,24,25].

### 2.4. ALAD Genotyping

Genomic DNA obtained from oral mucosa and collected by swab was extracted using an extraction kit (Qiagen) following the procedures recommended by the manufacturer. The genotyping analysis of *ALAD* (chr9:113391611) *177 C > G* (rs1800435) missense variant was performed using a *TaqMan* allelic discrimination assay (C_11495146_10) by 7500 Real-Time System (Applied Biosystems, Foster City, CA, USA), and the genotypes were directly determined. PCR amplification was performed in 8 μL reactions with 1 μL of template DNA (3–23 ng/μL), 1 × TaqMan Universal Master Mix, 1 × each primer, and probe assay. Thermal cycling was initiated with a first denaturation step of 10 min at 95 °C, followed by 40 cycles of denaturation at 92 °C for 15 s and annealing at 60 °C for 1 min. *ALAD* allele frequency and genotype distribution were derived by gene counting.

### 2.5. Data Analysis

Continuous variables were reported as mean ± standard deviation (SD), range, median, and interquartile range (IQR). Categorical data were shown as number (*n*) and frequency (%). Deviations from Hardy–Weinberg equilibrium (HWE) in *ALAD* polymorphism frequency were assessed by the goodness-of-fit χ2 test. The Kruskal–Wallis test was performed to evaluate the differences in Hg-levels between groups. All analyses were performed using the IBM SPSS 20.0 Statistics for Windows (SPSS Inc., Chicago, IL, USA).

## 3. Results

The present study comprised 103 children under 15 years old, from which 46 (44.7%) reside in *Sawré Muybu*, 38 (36.9%) in *Poxo Muybu,* and 19 (18.4%) in *Sawré Aboy* villages. The mean age of all indigenous children was 6.6 ± 4.5 years old and 62 (60.2%) were female. The anthropometric measurements are shown according to the age group: 29 (28.2%) were over 10 years old, 31 (30.1%) were between 6 to 10 years old, and 43 (41.7) children were between 0 to 5 years old (Table 1).

The rate of successful genotyping of the *ALAD C > G* was 100%, and the distribution of polymorphism was in Hardy–Weinberg equilibrium (*p*-value = 0.92). Of the 103 indigenous children, only two from *Sawré Muybu* village were heterozygous *ALAD CG*. Minor allele frequency (MAF) of *ALAD G*, wild-type (*ALAD CC*), and heterozygous (*ALAD CG*) frequency genotypes in all subjects was 0.97%, 98.1%, and 1.9%, respectively. Table 1 displays the sociodemographic and genetic characteristics of the studied population (*n* = 101) compared with the two children with *ALAD* polymorphism.

All indigenous children’s mean hair mercury concentration was 7.0 ± 4.5 (1.4–23.9) µg/g. The prevalence of mercury exposure above 6.0 µg/g was 49.0% in all studied population (*n* = 102) and 66.7% in children between 12 to 14 years old (*n* = 21). Both heterozygous (*ALAD CG*) genotype children from *Sawré Muybu* had mercury levels above the average and third interquartile of the studied population (*n* = 100), considering only *Sawré Muybu* or *Poxo Muybu* villages. In addition, the mercury levels of *ALAD CG* children were considered as a health risk indicator (≥6.0 µg/g). There were statistically significant variations between the three villages (Kruskal–Wallis = 17.9; *p*-value < 0.0001). The *Sawre Aboy* people showed the highest risk of mercury contamination compared with other villages (Table 2). One wild-type *ALAD* child from *Sawré Aboy* (10-year-old boy, BMI 17.2) showed the highest mercury concentration (23.9 µg/g), according to the high levels of the village. For all participants under 15 years old, the median level of mercury in girls (*n* = 61) was 5.35 µg/g (IQR: 3.93, 5.35 and 9.17; range 1.60–22.07) and in boys (*n* = 41) it was 6.12 µg/g (IQR: 4.28, 6.12 and 8.94; variation 1.42–23.87) (*p*-value = 0.835). Considering the participants between 12 to 14 years old, the median level of mercury in girls (*n* = 13) was 6.19 µg/g (IQR: 4.24, 6.19 and 10.55; range 2.00–16.44) and in boys (*n* = 8) it was 8.13 µg/g (IQR: 5.76, 8.13 and 8.96; variation 5.76–9.13) (*p*-value = 0.515).

Regarding blood pressure levels, 22 (21.4%) adolescents over 12 years old from whom we had this data, were classified as normal. Systolic blood pressure (SBP) mean level was 105 ± 6.8 (92–116), and diastolic blood pressure (DBP) mean level was 63 ± 6.8 (54–76). Peripheral blood samples were assessed for children from over 6 months old to 14-year-old adolescents (*n* = 99). For all participants, the hemoglobin levels vary from 9.2 to 16.1 g/dL (mean 12.4 ± 1.2). The prevalence of anemia (Hb level ≤ 11.5 g/dL) was 48.5% for all participants and 23.8% for adolescents between 12 to 14 years old (*n* = 21).

Table 3 displays the clinical characteristics and occurrence of mercury exposure symptoms of the studied population compared with two children with *ALAD* polymorphism. Both heterozygous genotype (*ALAD CG*) children had adverse neurological symptoms associated with chronic mercury exposure. The first case is a 12-year-old male that, from the 15 individuals able to be tested (aged 12–14 years old), was the only one that presented visual field alterations such as loss of differential light sensitivity and leukocoria in his right eye. In addition, the 14-year-old girl had the highest number of neurological manifestations (three of the four observed symptoms), including cognitive, somatosensory, and motor symptoms, such as memory deficit (evidenced by the verbal fluency test), distal neuropathy, and toe amyotrophy. The 12-year-old boy also reported recurrent diarrhea and acute respiratory infections, including an event of hospitalization for pneumonia. The boy’s mother reported that his birth weight was 2000 g. Both children reported frequent consumption of fish in the diet, at least three times a week. Regarding the age group and neurological tests, 56 children (54.4%) aged between 0 to 6 years old had Denver II test results. Amongst them, nine children had a risk for developmental impairment (Table 3), from which one presented three symptoms (*Poxo Muybu*), one presented two symptoms (*Sawré Muybu*), and the other seven presented only one symptom (4 *Sawré Muybu* and 3 *Sawré Aboy*). However, this children’s median mercury level was 6.2 µg/g (IQR: 3.2, 6.2, and 9.9), therefore, lower than in the two heterozygous *ALAD CG* children (Table 2).

## 4. Discussion

In this study, we described the effects of chronic exposure to mercury in 103 indigenous children living in the Brazilian Amazon, who face the burden of long-term impacts from illegal mining activities. Genetic analysis identified two children with the *ALAD* polymorphism who had high levels of mercury in their hair samples and severe symptoms of chronic mercury exposure. Although there is a growing number of studies investigating the effects of chronic exposure to mercury on population health [5,8,25,26], little is known about the influence of the individual’s genetic susceptibility on mercury toxicokinetics and toxidinamics, thus gaps remain in our knowledge about the role that genetic variability plays.

As far as we know, only one study has evaluated the effects of *ALAD* polymorphism on mercury-exposed individuals from the Brazilian Amazon. As previously described, heterozygous individuals *ALAD CG* had significant lower ALAD activity and higher Hg levels in blood compared with non-variant genotype subjects [11]. In the present study, both *ALAD CG* individuals had a mercury concentration even higher than the average, which suggests that the polymorphism is also contributing to mercury half-life time. The amino acid change caused by the *ALAD 177 C > G* polymorphism, resulted in a more negatively charged ALAD isozyme, which makes it more attracted to inorganic metals, such as lead (Pb) for example, and therefore, promotes higher levels of metal and free erythrocyte protoporphyrin in blood and tissues [27]. Since lead and mercury have similar atomic radii (1.76 and 1.81 Å, respectively) [28], and the ALAD enzyme may interact with other metals besides Pb [29,30], it is possible that Hg is capable of binding to the same sites in ALAD, also resulting in higher Hg levels in total blood [11]. *ALAD* polymorphisms and levels of essential (iron, copper, selenium, and manganese) and toxic trace elements (lead, cadmium, Hg, and arsenic) were tested in a Kyrgyz-stan population of uranium legacy sites. Lead blood variability was associated with *ALAD* polymorphisms with a slight influence on Hg, arsenic, and selenium levels also observed [31].

The average concentration of hair mercury in all studied population exceeds the reference limit (≥6.0 µg/g), which is in agreement with other studies from the Amazon region [24,25,32]. Recently our group evaluated Hg levels of 197 hair samples (ranging from 1.4 to 23.9 µg/g) and observed significant differences between the three villages (*Sawré Muybu*, *Poxo Muybu* and *Sawré Aboy*), with the residents of *Sawre Aboy* village at highest risk of mercury contamination [14], according to the present study. Once ingested through contaminated food, the digestive tract easily absorbs MeHg and it reaches the bloodstream, affecting various tissues in the human body, including the nervous system, due to its highly lipophilic nature [33]. In pregnant women, MeHg has the capacity to cross the placental barrier and to contaminate breast milk, exposing both fetuses and infants to this toxic compound [34,35].

The higher possibility of absorption through the digestive tract and the immaturity of the blood–brain barrier and metabolic excretory pathways, makes children one of the demographics most vulnerable to the effects of mercury exposure [13,36]. In line with this, children have a higher susceptibility to adverse neurological symptoms caused by chronic mercury exposure compared to adults with similar levels [7]. Chronic mercury exposure can cause serious neurological damage such as decrements in memory, attention, language, and visual-motor skills in childhood [13,33]; this is in accordance with what was observed in our present study, especially with the two children with the heterozygous *ALAD CG* genotype, who had a high risk of mercury contamination. *ALAD* polymorphism may modulate the burden of mercury exposure in the body and, consequently, induce metal toxicity [11]. Despite little evidence, Santos-Lima et al. (2020) identified in 263 children aged 6–14 years old from the Amazon region, several changes in neuropsychological functions, related to mercury exposure [13]; however, the children’s genetic profile was not evaluated.

There is evidence that maternal-fetal genetic background can modulate fetal exposure to these neurotoxicants [37]; however, studies focused on the association between Hg exposure and *ALAD* polymorphism are very scarce. From the 103 children in our study population, two presented *ALAD* polymorphism. Both these children were the same children that presented the most significant burden of neurological and clinical abnormalities, including toe extensor amyotrophy, distal polyneuropathy, cognitive and memory deficits, visual field changes, and leukocoria. Although the symptoms were different between the two cases, both children shared genetic backgrounds, had very high levels of mercury, and resided in the same village. *Sawré Muybu* village presented the lowest average mercury level and, therefore, had the same environmental exposure to mercury. Since it was the village with the lowest level of mercury and cases exceed the reference limit (≥6.0 µg/g), we hypothesize that the presence of the variant *ALAD* allele influences the occurrence of these disturbances by increasing the association between ALAD protein and Hg molecules and, consequently, raising blood Hg levels. Nevertheless, in addition to the *ALAD* polymorphism, there may be other genetic influences, making individuals more predisposed to certain conditions influencing the occurrence of neurological symptoms [38,39,40,41,42]. Visual field defects, for instance, have already been associated with a mutation on the *OPA 1* (*OPA1 Mitochondrial Dynamin Like GTPase*) gene [39] and a polymorphism near the *TGFBR3* (Transforming Growth Factor Beta Receptor (3) gene [38]. Another example is the *COMT* gene (*Catechol-O-Methyltransferase*), whose SNPs have been associated with lower chances of developing memory impairment [41] and higher odds of developing distal neuropathic pain [40]. A review study published in 2020 showed that epigenetic alterations such as miRNA expression and DNA methylation could cause impaired memory and neuronal development, depression-like behavior, visual deficits, and hyperactivity, in experimental models [42].

In addition, several studies have investigated the influence of mercury exposure on visual function [43,44,45] and sensorimotor polyneuropathy [46], symptoms here that were observed in the child with the *ALAD* polymorphism. A recent study, conducted in two Amazonian populations, investigated the visual functions of riverine individuals exposed to MeHg through food intake. The authors observed that all riverine subjects had a perimetric area smaller than the reference value and loss in color vision [45]. Another study, involving children from Turkey, showed that, besides the visual field defects, peripheral neuropathy was also a consequence of acute mercury exposure [43]. As previously described, mercury can cross the blood–retina barrier, accumulating in the cells and layers of the retina, inducing changes in the photoreceptors and, consequently, causing visual field defects [47].

Another neuropsychological function that can be disrupted for mercury exposure is the memory [48,49], also observed in one of the studied children with *ALAD* polymorphism. Grandjean and cols. (2014) conducted a cohort study with 694 individuals born in 1986 and 1987 in the Faroe Islands, investigating the association between postnatal exposures to MeHg and neurobehavioral performance at the age of 7. The study demonstrated that, from the 17 supposed outcomes, only the visuospatial memory measure showed a clear negative association after the adjusted analysis [49]. Furthermore, there is evidence that mercury exposure can also be a cause of Alzheimer’s disease [50]. A recent study reported a case of an old man diagnosed with Alzheimer’s disease, presenting with high levels of mercury due to MeHg-containing fish consumption. After a detoxification dietary regime, his body mercury levels dropped and his memory partially improved [51].

Almost half of the studied population had anemia (Hb level ≤ 11.5 g/dL). There is little evidence in the literature regarding this matter [26], however, a few recent studies showed an inverse association between hemoglobin levels and mercury exposure in the last decade [52,53,54]. A study from 2017, conducted with 83 children from the Peruvian Amazon region, demonstrated that MeHg exposure was associated with toxicant-induced anemia [54]. Nevertheless, Hb levels of two *ALAD* polymorphism children were within the normal range. Regarding the blood pressure, both SBP and DBP in the present study were found to be normal considering all indigenous children or specifically the *ALAD CG* individuals. High concentrations of Hg may play a role in blood pressure measurements since subclinical vascular changes start at an early age, even though studies investigating the relationship between Hg exposure and altered blood pressure provide conflicting results [55]. In addition, it is important to highlight the complexity of identifying effects related to long-term Hg exposure due to the absence of accurate diagnosis and the ability of Hg to deposit in different parts of the body, modifying many molecular pathways [7].

Within the Brazilian population, the MAF of *ALAD 177 C > G* polymorphism is low (approximately 1–7%) [56,57,58], with the majority being found in riverside communities of the Brazilian Amazon (2.7%) [11], an observation also seen in the present study, with only two subjects being heterozygous for the *ALAD CG* genotype (1.9%). Although an obvious limitation was the small sample size, the strengths of this study included the accurate analyses of the indigenous children’s genetic profile and individual mercury exposure levels alongside thorough assessment of neuropsychological function, general health status, and the sociodemographic characterization of the families. The results can be used to build a database from different populations chronically exposed to mercury in order to help identify the health impacts of illegal mining activities.

## 5. Conclusions

Despite the low frequency of *ALAD 177 C > G* polymorphism in the study population, both children with the heterozygous *ALAD* genotype presented with the health risk indicators of high mercury concentration levels, exceeding the reference limit, and severe neurological symptoms associated with chronic mercury exposure. It is worth mentioning that this is the first time an analysis of genetic polymorphism, focusing on mercury toxicokinetics and toxicodynamics, has been carried out among an indigenous children population living in remote areas of the Brazilian Amazon. Therefore, the knowledge regarding possible individual predisposing factors to the effects of heavy metals exposure is essential to understand the health situation of a hard-to-reach population and to inform public policy to help mitigate the extensive impact of gold mining activity in exposed communities within the Amazon region.

## Figures and Tables

**Table 1:** Epidemiological features and *ALAD* genotypes of the study population (*n* = 103), *Sawré Muybu* Indigenous Land, Pará, Amazon, Brazil, 2019.

Variables	Case 1	Case 2	Children Population *n* = 101 (%)
Munduruku indigenous group	*Sawré Muybu*	*Sawré Muybu*	*Sawré Muybu*, *n* = 44 (43.6%) *Poxo Muybu*, *n* = 38 (37.6%) *Sawré Aboy*, *n* = 19 (18.8%)
Sex	Male	Female	Female, *n* = 61 (60.4%) Male, *n* = 40 (39.6%)
*ALAD* genotypes	*CG*	*CG*	Wild-type (*CC*)
			**Mean ± SD (range) median**
Age (years)	12	14	6.4 ± 4.4 (0–14) 6.0 IQR = 3; 6 and 10
BMI (Over 10 years old) ^a^	18.9	21.7	18.8 ± 1.9 (15–22) 18.9 IQR = 17; 19 and 20
BMI (6 to 10 years old) ^b^	-	-	16.0 ± 1.3 (14–22) 15.6 IQR = 15; 16 and 17
Anthropometric measurements (0 to 5 years old) ^c^	-	-	0.16 ± 0.8 (−1.7–1.8) 0.17 IQR = −0.4; 0.2 and 0.8

BMI: Body mass index (Kg/m^2^). SD: standard deviation. IQR: interquartile range. ^a^ Data were obtained from 29 children over 10 years old. ^b^ Data was obtained from 31 children between 6 to 10 years old. ^c^ Z-score was obtained from 42 children between 0 to 5 years old (missing *n* = 1).

**Table 2:** Hair mercury levels (µg/g) in the study population (*n* = 102) *Sawré Muybu* Indigenous Land, Pará, Amazon, Brazil, 2019.

Variables	Case 1	Case 2	Children Population (*n* = 100) Mean ± SD (Range) Median
Mercury (µg/g) ^a^	11.8	9.1	7.0 ± 4.5 (1.4–23.9) 5.8 IQR = 4.0; 5.8 and 8.8
	*Sawré Muybu* ^b^ (*n* = 45)	*Poxo Muybu* (*n* = 38)	*Sawré Aboy* (*n* = 19)
Mercury (µg/g) ^c^	5.9 ± 4.5 (1.6–22.1) 4.4 IQR = 3.2; 4.4 and 7.3	6.3 ± 2.5 (1.4–11.8) 6.4 IQR = 4.5; 6.4 and 7.9	10.9 ± 5.6 (2.6–23.9) 10.1 IQR = 6.1; 10.1 and 15.2

IQR: interquartile range. ^a^ Data were obtained from 102 children. ^b^ Data were obtained from 44 children. ^c^ Significant different between the three villages (Kruskal–Wallis = 17.9; *p*-value < 0.0001).

**Table 3:** Clinical characteristics of the study population *Sawré Muybu* Indigenous Land, Pará, Amazon, Brazil, 2019.

Variables	Case 1	Case 2	Children Population Mean ± SD (Range) Median
Blood pressure ^a^ SBP (mmHg)	107	112	105 ± 6.9 (92–116) 106 IQR = 98; 106 and 110
DBP (mmHg)	69	73	62 ± 6.6 (54–76) 61 IQR = 56; 61 and 69
Hemoglobin level (g/dL) ^b^	14.8	14.2	12.4 ± 1.2 (9–16) 12.3 IQR = 11.8; 12.3 and 13.1
Symptoms of chronical mercury exposure ^c^			(*n* = 13)
Visual Field	Yes	No	Yes (*n* = zero)
Toe extensor amyotrophy	No	Yes	Yes (*n* = 2)
Distal neuropathy	No	Yes	Yes (*n* = 3)
Cognitive deficit	No	Yes	Yes (*n* = 4)
Denver neurodevelopment test ^d^			*n* = 56 (%)
Deficit language	-	-	8 (14.3)
Fine motor skills	-	-	2 (3.6)
Gross motor skills	-	-	2 (3.6)

SBP: systolic blood pressure. DBP: diastolic blood pressure. IQR: interquartile range. ^a^ Data were obtained from 22 children over 12 years old. ^b^ Data were obtained from 99 children. ^c^ Data were obtained from 15 children aged 12 years or older. ^d^ Data were obtained from 56 children aged between 0 to 6 years old.

## Data Availability

Data sharing not applicable.
